# Preparation and Performance Study of n-Undecane Phase Change Cold Storage Material

**DOI:** 10.3390/ma17071570

**Published:** 2024-03-29

**Authors:** Luchao Yan, Yang Wang, Shijian Lu, Zhipeng Zhu, Lingling Xu

**Affiliations:** College of Material Science and Engineering, Nanjing Tech University, Nanjing 211816, China; 202161203204@njtech.edu.cn (L.Y.); 202261103012@njtech.edu.cn (Y.W.); 202161103065@njtech.edu.cn (S.L.); 202161103006@njtech.edu.cn (Z.Z.)

**Keywords:** phase change microcapsules, n-undecane, suspension polymerization, cold storage

## Abstract

With the fast development of the cold chain transportation industry, the traditional refrigeration method results in significant energy consumption. To address the national call for energy saving and emission reduction, the search for a new type of energy storage material has already become a future development trend. According to the national standard GB/T28577 for the classification and basic requirements of cold chain logistics, the temperature in frozen logistics is typically below −18 °C. In this study, n-undecane with a phase change temperature of −26 °C is chosen as the core material of microcapsules. Poly(methyl methacrylate) is applied as the shell material, with n-undecane microcapsules being prepared through suspension polymerization for phase change cold storage materials (MEPCM). Using characterization techniques including SEM, DSC, FTIR, and laser particle size analysis, the effects of three types of emulsifiers (SMA, Tween-80, Tween-80/span-80 (70/30)), SMA emulsifier dosage, core–shell ratio, and emulsification rate on the thermal performance and micro-surface morphology of n-undecane/PMMA microcapsules were studied. The results indicate that when comparing SMA, Tween-80, and Tween-80/span-80 (70/30) as emulsifiers, the dodecane/PMMA microcapsules prepared with SMA emulsifier exhibit superior thermal performance and micro-surface morphology, possessing a complete core–shell structure. The optimal microstructure and the highest enthalpy of phase change, measuring 120.3 kJ/kg, are achieved when SMA is used as the emulsifier with a quantity of 7%, a core-to-wall ratio of 2.5:1, and an emulsification speed of 2000 rpm. After 200 hot and cold cycles, the enthalpy of phase change decreased by only 18.6 kJ/kg, indicating the MEPCM thermal performance and cycle life. In addition, these optimized microcapsules exhibit favorable microstructure, uniform particle size, and efficient energy storage, making them an excellent choice for the refrigeration and freezing sectors.

## 1. Introduction

Since entering the new century, the energy supply has been very tense in China. Redevelopment and rational use of energy have become a vital topic of common concern to all countries. In the various consumptions of energy, cold chain logistics will undoubtedly occupy a certain proportion [1]. Cold chain refers to a special logistics system that processes, stores, transports, sells, and distributes temperature-sensitive goods harvested from the place of origin until the consumer, aiming to ensure that each stage of the process is always under the required low-temperature conditions, to adequately ensure product quality and safety, to lower breakage, as well as to prevent loss [2,3,4]. Phase change cold storage technology is a significant tool for peak shifting and valley filling in power grid operations. As research continues to deepen, the successful development of novel phase change materials for cold storage has been realized. This aligns with the contemporary theme of energy conservation and environmental protection. Applying this technology in temperature-controlled packaging and cold chain transportation holds significant potential, thereby underlining its paramount importance [5,6,7,8]. Phase change cold storage materials are mainly divided into sensible heat energy storage (solid or liquid specific heat energy storage) materials, semi-latent heat storage (chemical reaction energy storage) materials, and latent heat energy storage (material phase change energy storage) materials of three kinds [9,10], of which the latent heat cold storage with its principle of simplicity, flexibility in design, the advantages of easy to use to become the market is currently the most extensively used a cold storage method. According to the phase change mechanism, phase change cold storage materials can be categorized into four categories: solid–solid, solid–gas, liquid–gas, and solid–liquid [11]. Among them, solid–liquid phase change cold storage materials mainly include inorganic hydrated salts, metal alloys, advanced aliphatic hydrocarbons, fatty acids, aliphatic alcohols, polymers, etc. [12], which have become a research hotspot in recent years owing to their advantages including small volume change before and after phase change, large latent heat of phase change, a wide range of phase change temperatures, and good stability [13]. In the current technology, the phase change temperature of solid–liquid phase change cold storage materials is mostly above −18 °C, and there are common problems including volume change during phase change, poor thermal conductivity in solid state, easy leaks in liquid state, and easy phase separation in long-term use. In addition, microencapsulation of phase change materials can be a good solution to these problems [14,15].

The encapsulation principle of microencapsulated phase change material is to encapsulate the phase change material in the shell material formed by polymer, and thus the phase change material will not be leaked to the external environment during the phase change; thus, the microencapsulated phase change material can be approximated as a solid substance that will not change its form [16]. The phase change material is used as the core material and the polymer is applied as the outer shell material of the microcapsule. With the external ambient temperature being different from the phase change temperature of the phase change material, the phase change material will undergo a phase transition, and it will regulate the external temperature to keep the same temperature as the phase change temperature by absorbing or releasing heat [17,18,19,20]. The role of the shell material is to prevent the phase change material from leaking during the phase transition process, which not only prevents the phase change material from leaking and polluting the environment but also makes the phase change material recyclable and sustainable [21]. Common microcapsule preparation methods include in situ polymerization [22,23], interfacial polymerization [24,25], complex coalescence [26,27], and suspension polymerization [28,29,30,31]. For example, Ikutegbe et al. [32]. used commercially available pure temperature P6 (peak melting point = 6.2 °C, ΔH = 150.1 kJ/kg) and crosslinked polymethyl methacrylate as the PCM and shell materials, respectively. Photoinduced suspension polymerization technique was used to package low melting point PCM in a UV perfluoroalkoxy (UV PFA) coil reactor. The effects of different synthesis parameters such as polymerization time and optimum core–shell mass ratio on the synthesis were investigated. The results show that when the polymerization time is 20 min and the core–shell mass ratio is 2:1, the peak melting temperature of the prepared microencapsulated PCM (m-PCM) is 8.2 °C, the average latent heat is 131.1 kJ/kg, and the content of PCM is 87.4%. After many heating/cooling cycles, m-PCM shows good thermal reliability, indicating that it has good fault tolerance. Wu et al. [33]. prepared thermochromic microencapsulated phase change materials (TC-MPCMs) for the development of pharmaceutical cold chain logistics. In the paper, TC-MPCMs for low temperature were constructed by encapsulating crystal violet lactone (CVL), bisphenol A (BPA), and n-tetradecane and n-dodecanol (aliphatic compound) binary system into melamine–formaldehyde (MF) resin. The experimental results show that TC-MPCMs with 25 wt% 12OH and core/shell of 1.85 demonstrate excellent properties in terms of encapsulation efficiency, melting enthalpy, and color difference, which are 67.80%, 169.90 J/g, and 23.39, respectively. Synthesized TC-MPCMs present outstanding thermal durability during 100 thermal cycles and have appreciable thermal stability at temperatures not exceeding 96 °C. The vaccine refrigerator was designed to maintain a low temperature (2–8 °C) for 12.8 min. Hajba-Horvath et al. [34] developed the encapsulation of octyl laurate phase change material (PCM) by calcium alginate microencapsulation technology for environmentally friendly low-temperature energy storage. Calcium alginate shells are functionalized by Ag nanoparticles. Based on these values, 71.0 and 69.0% maximal PCM content in the microcapsules were determined by the differential scanning calorimetry method. Both the Ag-loaded and unloaded calcium alginate-octyl laurate PCM capsules maintained the high heat storing capacity after 250 warming and cooling cycles, which proved they did not suffer from leakage after the accelerated thermal test.

Research on microencapsulation of low melting point phase change materials for refrigeration applications has been scarce so far, with most studies focusing on temperatures above 0 °C. This study is based on the search for a microencapsulated phase change cold storage material that can be used for freezing and cold storage with a phase change temperature below −18 °C. With n-undecane as the core material and polymethylmethacrylate as the shell material, n-undecane/PMMA microencapsulated phase change materials were prepared by suspension polymerization, and the types of emulsifiers, and emulsifier dosage were investigated by characterization means including SEM, DSC, FTIR, and laser particle size analysis. SEM, DSC, FTIR, and laser particle size analysis were used to investigate the effects of emulsifier type, emulsifier amount, core-to-wall ratio, and emulsification speed on the thermal properties and microscopic surface morphology of n-undecane/PMMA microcapsules. The microcapsules prepared with the optimized process parameters have the advantages of high encapsulation efficiency and good microscopic morphology. These are ideal materials for cold chain transport refrigerant accumulators.

## 2. Experimental Section

### 2.1. Materials

n-Undecane (C_11_H_24_, CP, 98%) with a phase transition temperature of −26 °C and an enthalpy of phase transition of 144 kJ/kg was purchased from McLean Biochemicals Co. Ltd. in Shanghai, China. Methyl methacrylate (MMA, C_5_H_8_O_2_, CP, 99.5%) was purchased from Shanghai Lingfeng Chemical Reagent Co. Ltd. in Shanghai, China. Pentaerythritol triacrylate (PETRA, C_14_H_18_O_7_, AR, 96%) was purchased from Aladdin Biochemical Technology Co., Ltd., Shanghai, China. Styrene/maleic anhydride copolymer (SMA, CP, 80%) was purchased from Shanghai Leather Chemical Factory, Shanghai, China. In addition, azobisisobutyronitrile (AIBN, C_8_H_12_N_4_, CP, 98%) was purchased from Test Four Dimensions Chemical Co., (Shanghai, China).

### 2.2. Preparation of MEPCMs

**Figure 1:**
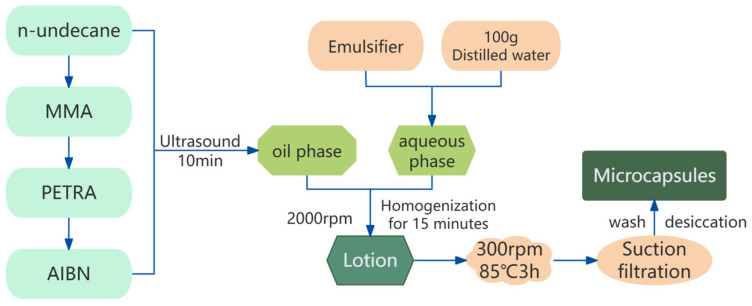
Flowchart of preparation of n-undecane phase change material microcapsules.

The microcapsules of n-undecane/poly(methyl methacrylate) phase change material were prepared by suspension polymerization method. The preparation flow chart is shown in Figure 1. To form the oil phase, a certain mass of n-undecane, methyl methacrylate, PETRA, and AIBN were weighed and ultrasonically mixed in a beaker, while a certain mass of distilled water and emulsifier was weighed and stirred until clarified to form the aqueous phase. The oil phase and aqueous phase were mixed at 50 °C with stirring at 2000 rpm for 15 min, then warmed up to 85 °C and stirred under nitrogen atmosphere at a constant speed of 300 rpm for 3 h. After the completion of reaction, the product was filtered with a vacuum pump, rinsed with anhydrous ethanol 2–3 times, and then rinsed again with distilled water 2–3 times to remove the unreacted MMA and the unencapsulated n-undecane. Finally, the product was dried in the oven at 45 °C to obtain the phase change material microcapsules.

### 2.3. Characterizations

#### 2.3.1. Microscopic Surface Morphology

The phase change material microcapsule particles were pasted on the conductive adhesive, and vacuum sprayed with gold for 2–5 min. The microscopic surface of the phase change material microcapsules was analyzed by scanning electron microscope (SEM) with JSM-5900 (TA Instruments, New Castle, DE, USA).

#### 2.3.2. Differential Scanning Calorimetry (DSC)

A model 404 F1 Pegasus differential scanning calorimetry was employed to analyze the thermal properties of the phase change microcapsules (Selb, Germany), with a temperature interval of −70 °C to 20 °C during the test, and heating or cooling cycles at a rate of 10 °C/min under nitrogen atmosphere.

#### 2.3.3. Fourier Transform Infrared Spectroscopy (FTIR)

To form a transparent sheet, 1–2 mg of the sample was ground with 200 mg of KBr, placed in a mold, and pressed in a hydraulic press, which was tested using a Scientific Nicolet iS5 Fourier Transform Infrared Spectrometer (FTIR) (Madison, WI, USA) with the number of scans set to 32, a resolution of 4 cm^−1^, and a range of wavelengths from 400 to 4000 cm^−1^.

#### 2.3.4. Particle Size Distribution

The MEPCMs were explored using a Malvern Mastersizer 2000 laser particle size analyzer with a range of 0.01–3000 μm (Malvern, UK). Prior to the experimental analyses, the microencapsulated powder needed to be diluted ultrasonically with an appropriate amount of distilled water.

#### 2.3.5. Encapsulation Ratio

The encapsulation rate is determined by the ratio of the latent heat of phase change in the microencapsulated PCM to the latent heat of phase change in the core material. It indicates that the larger the ratio, the higher the encapsulation rate, which can be calculated by the following formula:

$$W=\frac{∆H_{MEPCM}}{{∆H}_{PCM}}\times 100\%$$

where ΔH*_MEPCM_* represents the latent heat value of the microencapsulated PCM (kJ/kg), and ΔH_PCM_ is the latent heat value of the core PCM (n-undecane: 144 kJ/kg).

## 3. Results and Discussion

### 3.1. Effect of Emulsifiers on Phase Change Materials of n-Undecane Microcapsules

#### 3.1.1. Effect of Different Emulsifiers on the Microscopic Morphology of n-Undecane Microencapsulated Phase Change Materials

In this study, three different emulsifiers, SMA, Tween-80, and Tween-80/span-80 (70/30) were selected for the preparation of n-undecane microencapsulated phase change materials with the raw material ratios, as shown in Table 1.

**Figure 2:**
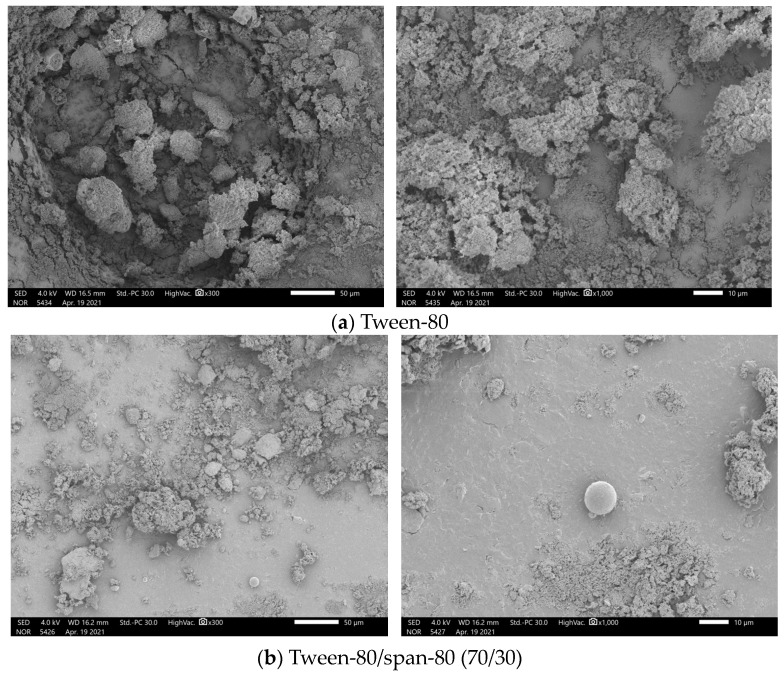

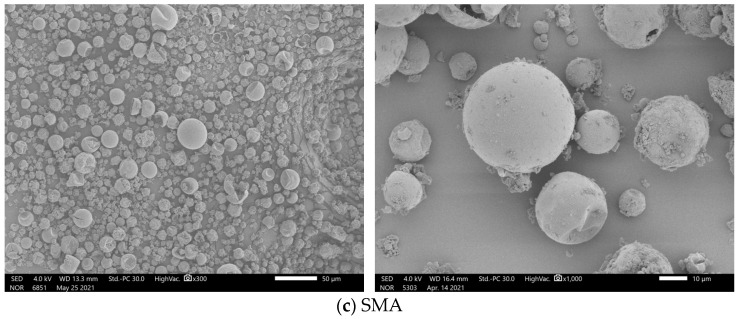
SEM image of n-undecanone microcapsule phase change cold storage materials prepared with different emulsifiers: (**a**) Tween-80; (**b**) Twain-80/Span-80 (70/30); (**c**) SMA.

Figure 2 shows the SEM of n-undecane microcapsules phase change cold storage materials prepared with three different types of emulsifiers. Figure 2a displays the SEM image of n-undecane/PMMA microcapsule phase change cold storage material prepared with Tween-80 as the emulsifier. Based on the SEM image, it can be found that the sample is in the form of loose sand, and there is no core–shell structure, which may be caused by the fact that the Tween-80, as a kind of non-ionic emulsifier, does not have a positive and negative charge in aqueous solution, and it is easily influenced by the system pH value and temperature during the reaction. With the increasing temperature in the process, the emulsion will produce an emulsion breaking phenomenon, which resulted in the poor emulsification effect, and the microcapsules could not be prepared.

When the emulsifier is the mixture of Tween-80 and Span-80 in the ratio of 7:3 (Tween-80/Span-80 = 70/30), the prepared n-undecane/PMMA microcapsule phase change cold storage material scanning electron microscope diagram as presented in Figure 2b, it can be observed that the sample has appeared in the phenomenon of agglomeration, with the structure of the core and shell of the microcapsule, but only accounts for a very small portion of the sample, and most of them are still n-undecane not encapsulated by PMMA, and the emulsification effect is not satisfactory.

Figure 2c shows the SEM image of n-undecane/PMMA microcapsule phase change cold storage material prepared with SMA as the emulsifier, from the SEM image. Clearly, the prepared sample has a complete core–shell structure of the microcapsule sphere. From the number of microcapsules or microcapsule phase appearance, n-undecane/PMMA microcapsule phase change cold storage material prepared by SMA emulsifier is better than the other two emulsifiers. Moreover, from the preparation process, there was neither foam nor emulsion breakage during the emulsification process using SMA as the emulsifier. Therefore, it can be found that SMA is the best emulsifier for the preparation of n-undecane/PMMA microcapsule phase change cold storage material among the three selected emulsifiers.

#### 3.1.2. Effect of the Dosage of Emulsifier on the Microscopic Morphology of n-Undecane Microencapsulated Phase Change Materials

With SMA as the emulsifier for the n-undecane microcapsule phase change material preparation system, the effect of the dosage of emulsifier SMA on the morphology of microcapsules was investigated. Table 2 shows the preparation material ratios for different emulsifier amounts.

**Figure 3:**
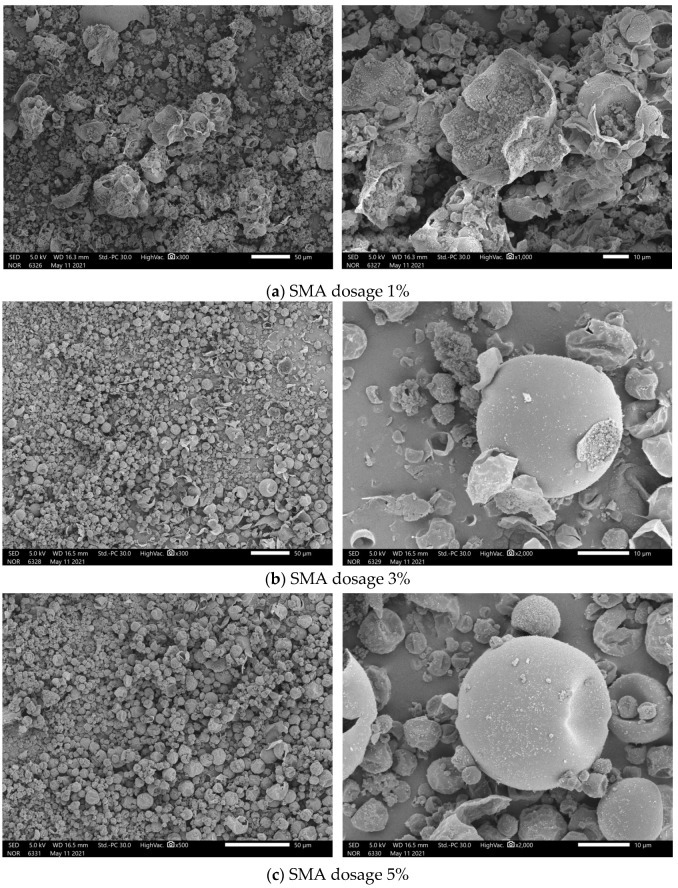
SEM images of n-undecanone microcapsule phase change cold storage materials prepared with different SMA dosages: (**a**) 1%; (**b**) 3%; and (**c**) 5%.

**Figure 4:**
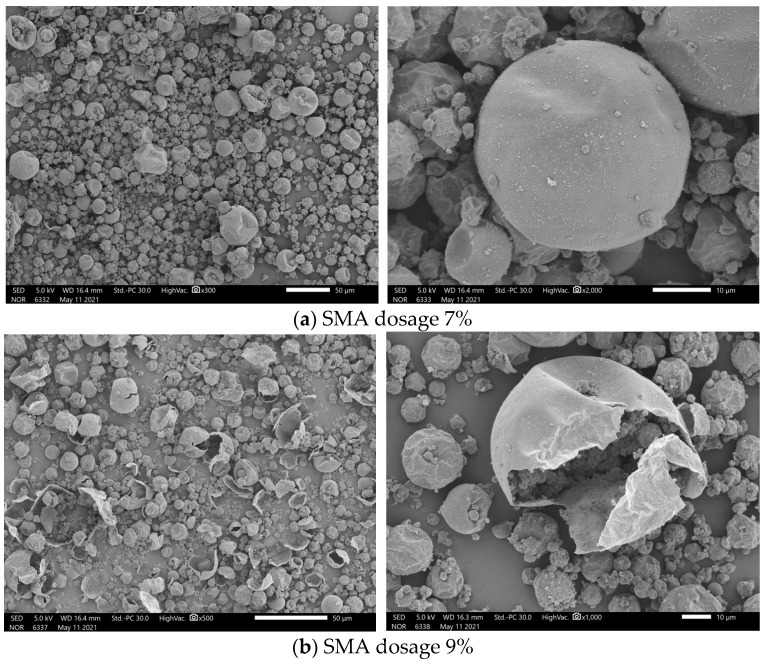
SEM images of n-undecanone microcapsule phase change cold storage materials prepared with different SMA dosages: (**a**) 7%; (**b**) 9%.

Figure 3 and Figure 4 show the SEM images of microcapsules prepared with different amounts of emulsifiers. The amounts of emulsifiers were 1%, 3%, 5%, 7%, and 9%, respectively. The results from the SEM images demonstrate that microcapsule spheres with core–shell structures appeared in the samples prepared with different amounts of emulsifier.

When the dosage of SAM was 1%, the SEM image is shown in Figure 3a. Although the presence of microcapsules can be observed in the sample, the degree of breakage is very large, and the vast majority of microcapsule balls exist in ruptured form. This is due to the fact that when the emulsifier content in the system is very low, the emulsification efficiency of the emulsion in the high temperature and high-speed emulsification process is very low, the stability of the emulsion is poor, and the efficiency of the self-polymerization of the shell monomers tends to decrease. Therefore, it is difficult for the shell to encapsulate the core material to form microcapsules.

With the increasing emulsifier dosage, as shown in Figure 3b,c, the number of microcapsule spheres of the samples prepared with 3% and 5% emulsifier dosage increased significantly, and the degree of microcapsule breakage was significantly reduced relative to that of the samples prepared with 1% emulsifier dosage. In addition, it can also be seen that the increase in emulsifier dosage from 1% to 5% is proportional to the improvement of microcapsule morphology.

Figure 4a,b show the SEM images of the samples prepared with SMA dosages of 7% and 9%, from which it can be found that when the SMA dosage was 7%, the surface morphology of the microcapsules was more regular compared to the previous ones, and fewer microcapsules were broken. However, when the SMA dosage was increased to 9%, the degree of broken microcapsules tended to suddenly increase. This may be caused by the fact that when the SMA is added in excess, there is an excess of SMA between the oil phase and the water phase, which produces a site-blocking effect. This leads to a reduction in the precipitation rate of the shell monomer during the polymerization process, which consequently decreases the amount of shell material encapsulated on the core material’s surface. As a result, in the post-reaction treatment process, the thinness of the shell material makes it susceptible to breakage. Consequently, the fragility of the shell increases the likelihood of microcapsule damage. This suggests that the addition of excess emulsifiers can also lead to the preparation of the microcapsules. The surface morphology was damaged.

From the SEM image, when the amount of SMA was increased from 1% to 7%, the microscopic surface morphology of the microcapsules improved with the increase in the emulsifier, while the continued increase in the amount of SMA led to the increase in the degree of breakage of the microcapsules. This was not conducive to the generation of microcapsules.

#### 3.1.3. Effect of Emulsifier Dosage on Thermal Properties of n-Undecane Microencapsulated Phase Change Materials

**Figure 5:**
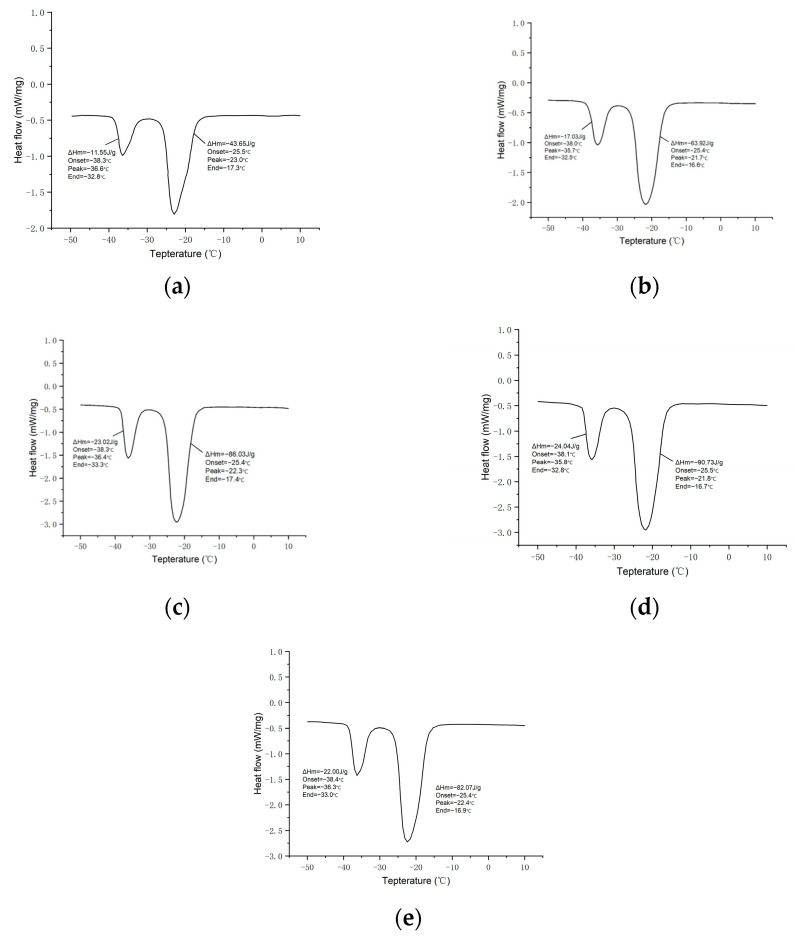
DSC images of n-undecanone microcapsule phase change cold storage materials prepared with different SMA dosages: (**a**) 1%; (**b**) 3%; (**c**) 5%; (**d**) 7%; and (**e**) 9%.

Figure 5 shows DSC diagrams of n-undecane microcapsule phase change materials prepared with different SMA dosages. The DSC results suggest two phase change peaks, one at approximately −22 °C and another at −36 °C. The enthalpy of the peak at −22 °C is significantly greater than the other, making it the primary energy storage temperature. Since the phase transition temperature of −22 °C aligns with the requirements for freezing and cooling storage, it can serve as the reference for calculating the enthalpy of phase transition in the n-undecane microcapsules.

Based on the DSC results, the data of phase change temperature and the enthalpy of phase change in n-undecane microcapsule phase change cold storage materials prepared with different SMA dosages are listed in Table 3, showing that the enthalpy of the phase change in microcapsules is the largest when the SMA dosage is 7%, and the highest encapsulation ratio is achieved, reaching 63%. The enthalpy of the phase transition of microcapsules increases with the increasing SMA addition from 1% to 7% and decreases when the SMA addition continues to increase to 9%. This is also consistent with the SEM results of microcapsules.

### 3.2. Effect of Core-to-Wall Ratio on Phase Change Materials of n-Undecane Microcapsules

#### 3.2.1. Effect of Core-to-Wall Ratio on the Microscopic Morphology of n-Undecane Microencapsulated Phase Change Materials

After determining the type of emulsifier and the amount of emulsifier as SMA and 7%, respectively, the effect of the core-to-wall ratio on the phase change material of n-undecane microcapsules was investigated. The process parameters of microcapsules prepared with different core-to-wall ratios are shown in Table 4.

**Figure 6:**
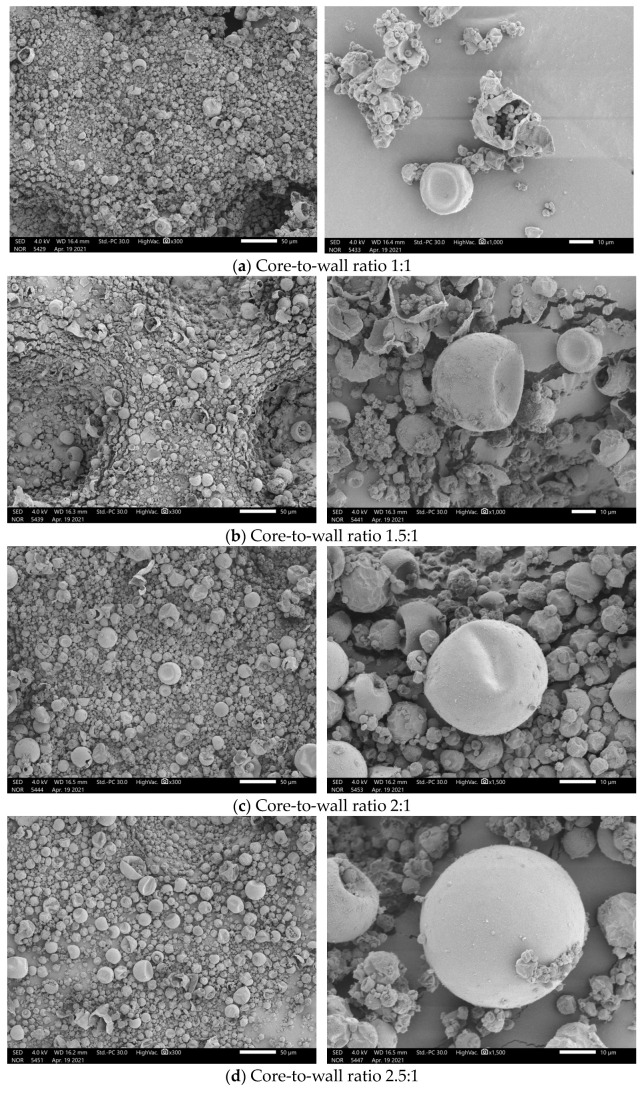

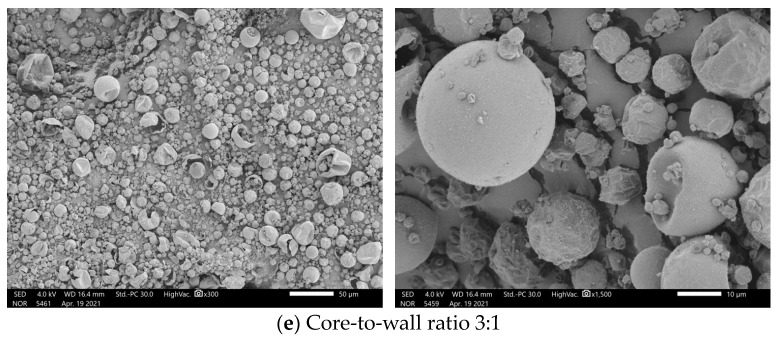
SEM images of n-undecane microcapsules phase change cool storage materials prepared with different core-to-wall ratios: (**a**) 1:1; (**b**) 1.5:1; (**c**) 2:1; (**d**) 2.5:1; and (**e**) 3:1.

Figure 6 shows the SEM images of n-undecane microcapsule phase change materials prepared with different core-to-wall ratios. The core-to-wall ratio is a vital parameter for the preparation of microcapsules, and the manifestation of microcapsules prepared with different core-to-wall ratios varies significantly. As can be seen from the SEM image, when the core-to-wall ratio is 1:1, the microcapsule spheres are mostly wrinkled and concave, which is caused by the fact that when the core-to-wall ratio is 1:1, there is relatively less core material in the system. This results in a lower quality of the core material encapsulated by the shell material, making it easy to form wrinkles and cavities in the generated microcapsules. With the increasing core-to-wall ratio, the core material in the system increases, and the surface folds and cavities of the microcapsules decrease. However, when the core-to-wall ratio increases to 3:1, the microcapsule ball begins to break. This is due to the amount of core material being too much, and the encapsulation capacity of the shell material exceeding the critical value, causing the shell material to become thinner, and it is easy to break, and fragmentation occurs.

Therefore, the core-to-wall ratio is not the bigger the better, and it is necessary to choose the appropriate core-to-wall ratio according to the actual situation.

#### 3.2.2. Effect of Core-to-Wall Ratio on the Thermal Properties of n-Undecane Microencapsulated Phase Change Materials

**Figure 7:**
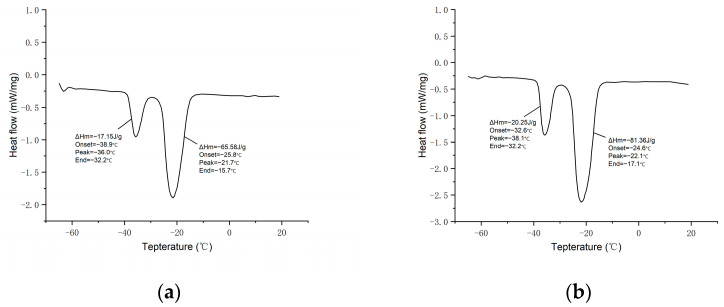

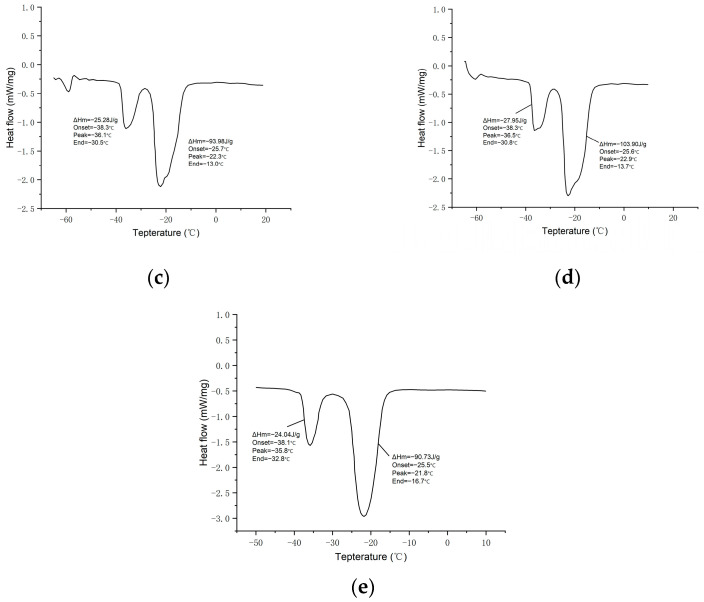
DSC diagram of n-undecane microcapsule phase change cold storage material prepared with different core wall ratios: (**a**) 1:1; (**b**) 1.5:1; (**c**) 2:1; (**d**) 2.5:1; and (**e**) 3:1.

Figure 7 shows the DSC plots of n-undecane microcapsule phase change materials prepared in accordance with different core-to-wall ratios, and the thermal performance parameters including phase change temperature and the enthalpy of the phase change in the n-undecane microcapsule phase change materials are detailed in Table 5.

Combined with the DSC plots and tabular data, when the core-to-wall ratio is 1:1, the phase transition enthalpy of n-undecane microcapsules is the smallest, which is 65.58 kJ/kg, and the encapsulation rate is 45.5%; moreover, when the core-to-wall ratio is 2.5:1, the phase transition enthalpy of n-undecane microcapsules is the largest, which is 103.9 kJ/kg, with the encapsulation rate reaching 65.3%. Obviously, when the core-to-wall ratio increases from 1:1 to 2.5:1, the coating rate of n-undecane microcapsules is directly proportional to the size of the core-to-wall ratio. This is caused by the fact that with the increasing core-to-wall ratio, the coated core material inside the n-undecane microcapsule increases, the enthalpy of the phase change in the microcapsule increases, and the coating rate of the n-undecane microcapsule naturally increases according to the formula of the coating rate.

However, when the core-to-wall ratio continued to increase to 3:1, the coating rate of n-undecane microcapsules tended to decrease rather than increase. Combined with the above-mentioned SEM results, this is due to the fact that when the core-to-wall ratio is too high, the shell material is not enough to encapsulate the core material. This causes the n-undecane microcapsule to be easy to break and the loss of the core material, resulting in a decrease in the enthalpy of the phase change, and a reduction in the coating rate naturally. Therefore, a high core-to-wall ratio is an obstacle to the preparation of microcapsules, and a core-to-wall ratio of 2.5:1 is the most suitable for the preparation of n-undecane microcapsules.

### 3.3. Effect of Emulsification Speed on Phase Change Materials of n-Undecane Microcapsules

#### 3.3.1. Effect of Emulsification Speed on the Microscopic Surface Morphology of n-Undecane Microencapsulated Phase Change Materials

The emulsification speed is the stirring rate of the phase change emulsion during the high-temperature emulsification reaction, which can directly influence the final particle size of the microcapsules and the microscopic surface morphology [35]. The effects of different emulsification speeds of 1250, 1500, 1750, and 2000 rpm on the morphology of microcapsules were set up with the emulsification speed as a one-way variable. The specific preparation parameters are shown in Table 6.

**Figure 8:**
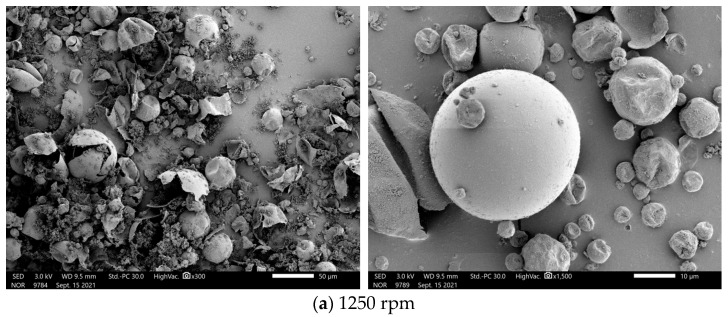

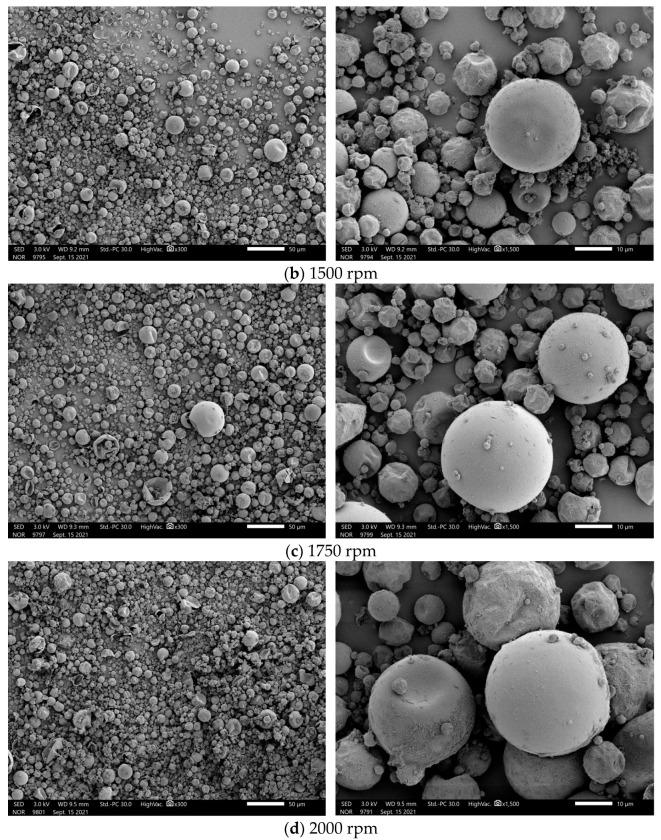
SEM images of n-undecane microcapsules phase change cool storage materials prepared at different emulsification speeds: (**a**) 1250 rpm; (**b**) 1500 rpm; (**c**) 1750 rpm; and (**d**) 2000 rpm.

Figure 8 shows the SEM images of n-undecane microencapsulated phase change materials prepared at four different emulsification speeds, namely 1250, 1500, 1750, and 2000 rpm. With the emulsification speed being 1250 rpm, as shown in Figure 8a, the sample has complete microcapsule spheres with core–shell structure, but most of the microcapsules are in the form of fragmentation, and the shell material seldom completely encapsulates the core material, which may be due to the emulsification speed being too low, and the provided shear stress during the emulsification reaction process is too small, resulting in the incomplete dispersion of the emulsion. Then, the core material of the organic material n-undecane alkane will be easy to form agglomerates, and not easy to coat with the polymerized shell material.

As the emulsification speed was increased to 1500 and 1750 rpm, as shown in Figure 8b,c, it can be observed that the number of intact microcapsules increased, and the surface was smoother, suggesting that the emulsion dispersed more uniformly during emulsification after increasing the emulsification speed. Under the same scale, it can also be found that the particle size of the microcapsules becomes smaller, and the particle size tends to be consistent.

When the emulsification speed reaches 2000 rpm, as displayed in Figure 8d, the particle size of microcapsules can be seen more concentrated, and it is concluded that the larger the emulsification speed, the smaller the particle size of microcapsules and the more concentrated the size of microcapsules; moreover, the microcapsule surface morphology is the best when produced at 2000 rpm.

#### 3.3.2. Effect of Emulsification Speed on the Thermal Properties of n-Undecane Microencapsulated Phase Change Materials

**Figure 9:**
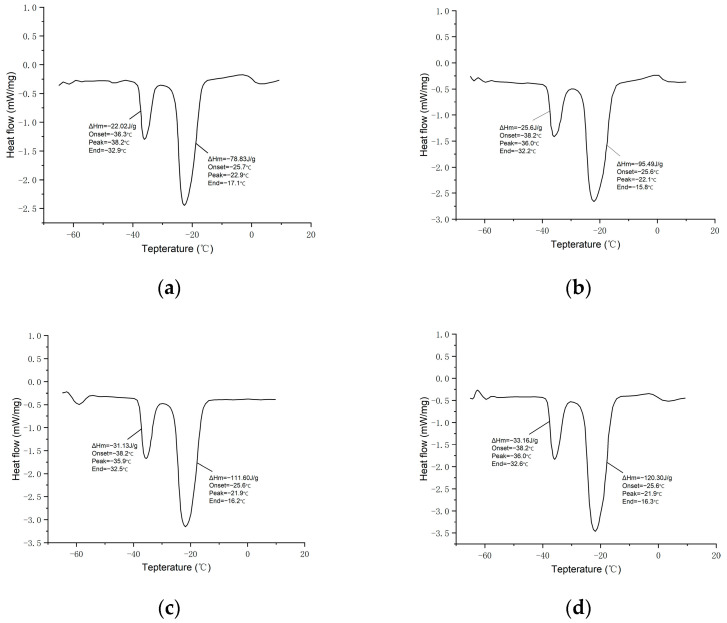
DSC images of n-undecane microcapsules phase change cool storage materials prepared at different emulsification speeds: (**a**) 1250 rpm; (**b**) 1500 rpm; (**c**) 1750 rpm; and (**d**) 2000 rpm.

Figure 9 shows the DSC plots of the phase change materials of n-undecane microcapsules prepared at different emulsification speeds, showing that the highest enthalpy of phase change in n-undecane microcapsules was 120.3 kJ/kg when the emulsification speed was 2000 rpm, and the encapsulation rate reached 83.3%. The composition and latent heat values of microcapsules used in previous cryogenic studies are shown in Table 7. PMMA-coated n-undecane has a higher enthalpy of phase change due to its uniform particle size, good sealing, and thus higher energy storage capacity of MEPCM than that of the previously investigated microcapsules. With the gradual increase in the emulsification speed from 1250 to 2000 rpm, the enthalpy of phase change in n-undecane microcapsules has been increasing, indicating that the higher the emulsification speed, the more thoroughly the emulsion is emulsified, the higher the encapsulation efficiency of the microcapsules, and the higher the enthalpy of phase change in the microcapsules.

In addition, it can be found from the data that the enthalpy of phase change in n-undecane microcapsules increased by 16.66 kJ/kg when the emulsification rotational speed was increased from 1250 to 1500 rpm; similarly, the enthalpy of phase change in n-undecane microcapsules increased by 16.11 kJ/kg when the emulsification rotational speed was increased from 1500 to 1750 rpm; finally, when the emulsification rotational speed was increased from 1750 to 2000 rpm, the enthalpy of phase change in n-undecane microcapsules increased by 8.7 kJ/kg, indicating that the trend of the increase in the enthalpy of phase change in n-undecane microcapsules was weakening with the increase in emulsification speed. Thus, it can be concluded that the emulsification speed should have a critical point, and should not be too fast, as the shear stress brought about by the emulsification speed is too rapid. Moreover, it may not be conducive to the formation of microcapsules.

#### 3.3.3. Particle Size Analysis of n-Undecane Microencapsulated Phase Change Materials

**Figure 10:**
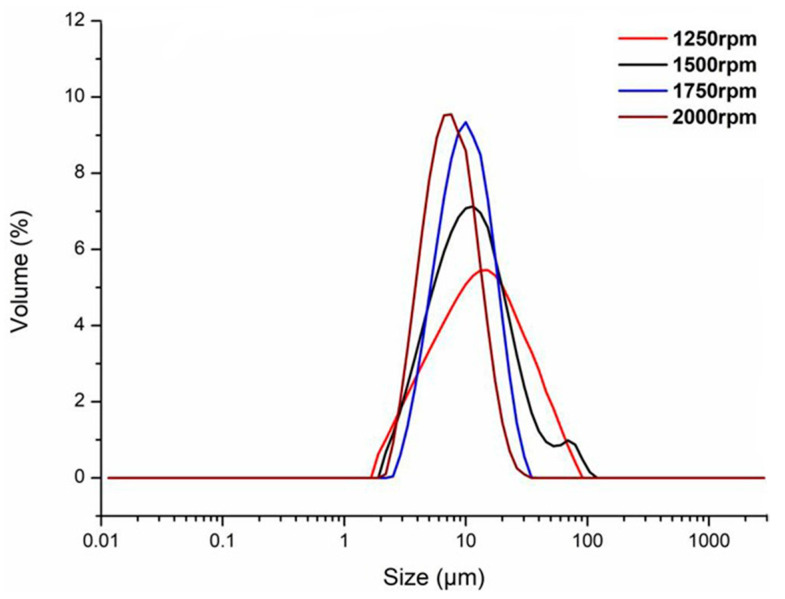
Particle size distribution curve of n-undecane microcapsules prepared at different emulsification speeds.

The particle size distribution curves of n-undecane microcapsules prepared at four different emulsification speeds, 1250, 1500, 1750 rpm, and 2000 rpm, are shown in Figure 10. The specific particle size parameters are listed in Table 8. We use D (0.5) to represent the average particle size of the microcapsules, Span is the breadth of the distribution interval of the microcapsules, and Uniformity is the uniformity of the particle size of the microcapsules. The average particle size of n-undecane microcapsules prepared at emulsification speeds of 1250, 1500, 1750, and 2000 rpm were 28.64, 22.47, 12.68, and 10.25 μm, respectively. It can be observed from the data of the particle sizes that the average particle size of microcapsules was reduced when the emulsification speed was increased from 1250 to 2000 rpm. In addition, it can be seen from the particle size data that when the emulsification speed was increased from 1250 to 2000 rpm, the average particle size of microcapsules decreased by 18.39 μm, and the average particle size of microcapsules decreased most when the emulsification speed was increased from 1500 to 1750 rpm, and the difference in the average particle size of microcapsules prepared by emulsification speeds of 1750 and 2000 rpm was relatively small. This demonstrates that when the emulsification speed is greater than 1500 rpm, the emulsification effect of the emulsion is significantly enhanced and then continues to increase the emulsification speed, and the average particle size of the microcapsules decreases to a smaller extent.

By combining the breadth of distribution of microcapsules and the uniformity of particle size distribution, when the emulsification speed was at 2000 rpm, the average particle size was 28.64 μm, Span = 1.68, Uniformity = 0.52, and the prepared microcapsules had the smallest particle size and the most uniform distribution of microcapsules.

### 3.4. FTIR Analysis of n-Undecane Microencapsulated Phase Change Materials

**Figure 11:**
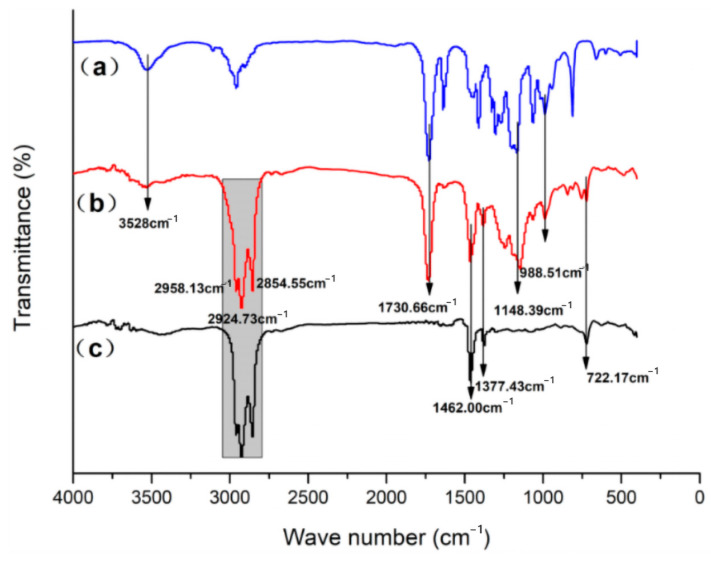
FTIR analysis spectrum (**a**) mma-petra polymer; (**b**) n-undecane/PMMA microcapsules; (**c**) n-undecane.

Figure 11 presents the FTIR spectral analysis of n-undecane microencapsulated phase change materials prepared according to the optimized process parameters.

Figure 11b shows the Fourier infrared (FTIR) spectra of n-undecane/PMMA microcapsule phase change material, while Figure 11a,c present the infrared (IR) spectra of the shell material PMMA and core material n-undecane, respectively. Based on the figures, the hydrocarbon stretching vibrational absorption peak of -CH=CH_2_ group at 3528 cm^−1^; the asymmetric stretching vibrational absorption peak of -CH_3_ group at 2958.13 cm^−1^; the asymmetric stretching vibrational absorption peak of -CH_2_ group at 2924.73 cm^−1^; the symmetric stretching vibrational absorption peak of -CH_2_ group at 2854.55 cm^−1^ and the symmetric stretching vibrational absorption peak of -CH_2_ group at 1730.66 cm^−1^. The stretching vibrational absorption peak of -C=O group at 1730.66 cm^−1^; the asymmetric deformation vibrational absorption peak of -CH_3_ group at 1462.00 cm^−1^; the asymmetric deformation vibrational absorption peak of -CH_3_ group at 1377.43 cm^−1^; and the carbon-hydrogen deformation vibrational absorption peaks at 988.51 cm^−1^ and 722.17 cm^−1^; peaks at 988.51 cm^−1^ and 722.17 cm^−1^. At the same time, these characteristic peaks of n-undecane/PMMA microcapsules matched those of n-undecane and PMMA, and no new characteristic peaks appeared, indicating that no chemical reaction was involved in the preparation of n-undecane/PMMA microcapsules and that it was purely a physical encapsulation between the core material and the shell material.

### 3.5. Recycle Stability Analysis

**Figure 12:**
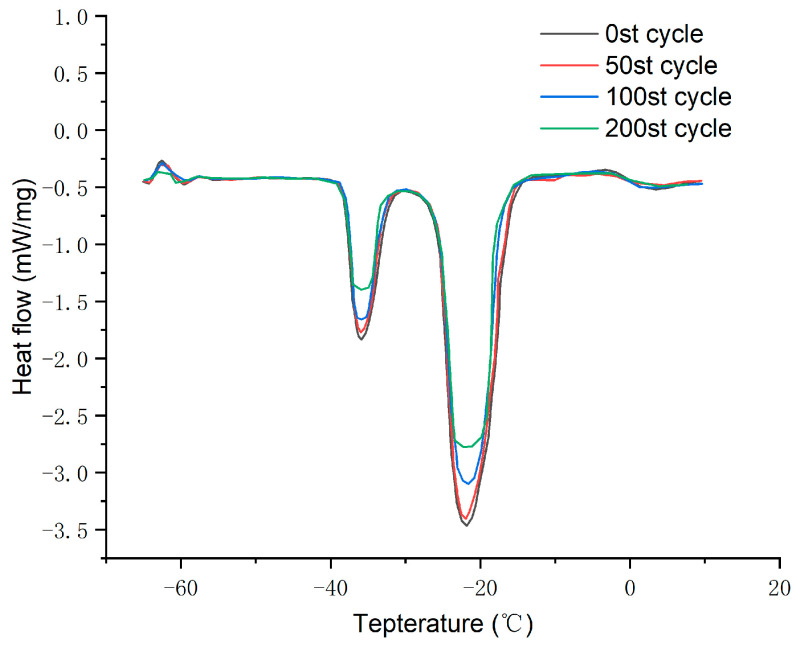
DSC curve of thermal cycle test of microencapsulated phase change material.

A certain amount of eicosane-based microencapsulated phase change material (MEPCM) was subjected to 50, 100, and 200 cold/hot cycles: each cycle involved heating at 20 °C for 10 min, followed by cooling at −50 °C for 10 min. Figure 12 displays the DSC curves of eicosane after three different numbers of cold/hot cycles, with specific thermal performance parameters listed in Table 9. The latent heat of fusion for MEPCM was 120.3 kJ/kg before undergoing any cold/hot cycles. These values decreased to 118.5 J/g after 50 cycles, 109.4 kJ/kg after 100 cycles, and further to 101.7 kJ/kg after 200 cycles. Compared to the material before any cycles, the enthalpy value decreased by 1.8 kJ/kg after 50 cycles, corresponding to a 1.2% reduction in encapsulation efficiency. After 100 cycles, the enthalpy value dropped by 10.9 kJ/kg, with a 7.6% decrease in encapsulation efficiency. Following 200 cycles, the enthalpy value decreased by 18.6 kJ/kg, and the encapsulation efficiency dropped by 12.9%. The data indicates that after 200 cycles, the encapsulation efficiency of MEPCM remains relatively stable, demonstrating its good thermal stability and cycle stability. Even after multiple thermal cycles, MEPCM can maintain a stable latent heat of fusion, indicating excellent thermal performance and cycle life of the phase change material. This underscores the crucial importance of the high reliability and stability of MEPCM in practical applications.

## 4. Conclusions

This study demonstrates the preparation and analysis of a phase change material microcapsule for cryogenic freezing. In this paper, n-undecane was used as the core phase change material and PMMA as the shell material, and the microencapsulated phase change material was prepared by suspension polymerization. Three emulsifiers, namely, SMA, Tween-80, and Tween-80/span-80 (70/30), were compared in the experiment. The results show that with Tween-80 as the emulsifier in the SEM image, the samples became loose sand and no core–shell structure appeared. With Tween-80/span-80 (70/30) as the emulsifier, the samples appeared agglomerated, with only a very small portion of them showing a core–shell structure, and most of them did not encapsulate n-undecane. When SMA was used as an emulsifier, the sample had a complete core–shell structure and PMMA successfully encapsulated n-undecane. When SMA was used as an emulsifier, the core–shell ratio was 2.5:1 and the amount of emulsifier was 7%. The n-undecane microcapsules prepared at an emulsification speed of 2000 rpm had the best microscopic morphology, a phase transition temperature of −21.9 °C, and a maximum enthalpy of the phase transition of 120.3 kJ/kg. FTIR analysis showed that there was no chemical reaction between the core material and the shell material to form a new substance, which was just a simple physical encapsulation. The phase change enthalpy of n-undecane microcapsules decreased by only 18.6 kJ/kg after 50, 100, and 200 hot and cold cycles, indicating that they have good thermal performance and cycle life. This study provides a new idea for the frozen logistics industry below −18 °C, and in-depth research on the application of phase change microcapsules in cold chain transport is of great practical significance and scientific value.

## Figures and Tables

**Table 1:** Diagram of raw materials for the preparation of microcapsules with different emulsifiers.

Types of Emulsifiers	Emulsifier Dosage (%)	Core–Wall Ratio	Crosslinking Agent Dosage (%)	Elicitor Dosage (g)
SMA	5	3:1	30	0.3
Tween-80	5	3:1	30	0.3
Tween-80/span-80(70/30)	5	3:1	30	0.3

**Table 2:** Raw material ratio of microcapsules prepared with different dosages of emulsifier.

Emulsifier Type	Emulsifier Dosage (%)	Core–Wall Ratio	Crosslinking Agent Dosage (%)	Elicitor Dosage (g)
SMA	1	3:1	30	0.3
SMA	3	3:1	30	0.3
SMA	5	3:1	30	0.3
SMA	7	3:1	30	0.3
SMA	9	3:1	30	0.3

**Table 3:** Thermal performance parameters of microcapsule phase change thermal storage materials prepared with different SMA dosages.

SMA Dosage (%)	Onset (°C)	End (°C)	Peak (°C)	Latent Heat (kJ/kg)	Encapsulation Ratio (%)
1	−22.5	−17.3	−22.0	43.65	30.3
3	−25.4	−16.6	−21.7	63.92	44.4
5	−25.4	−17.4	−22.3	86.03	59.7
7	−25.5	−16.7	−21.8	90.73	63.0
9	−25.4	−16.9	−22.4	82.07	56.9

Note: The enthalpy of phase change for pure n-undecane is 144 kJ/kg.

**Table 4:** Process parameters of microcapsule phase change thermal storage materials prepared with different core-to-wall ratios.

Core-to-Wall Ratio	Emulsifier Dosage (%)	Crosslinking Agent Dosage (%)	Emulsification Speed (rpm)	Elicitor Dosage (g)
1:1	7	30	1500	0.3
1.5:1	7	30	1500	0.3
2:1	7	30	1500	0.3
2.5:1	7	30	1500	0.3
3:1	7	30	1500	0.3

**Table 5:** Thermal performance parameters of microcapsule phase change thermal storage materials prepared with different core-to-wall ratios.

Core-to-Wall Ratio	Onset (°C)	End (°C)	Peak (%)	Latent Heat (kJ/kg)	Encapsulation Ratio (%)
1:1	−25.8	−15.7	−22.0	65.58	45.5
1.5:1	−24.6	−17.1	−22.1	81.36	56.5
2:1	−25.7	−13.0	−22.3	93.98	59.7
2.5:1	−25.6	−13.7	−22.9	103.9	65.3
3:1	−25.5	−16.7	−21.8	90.73	63.0

**Table 6:** Preparation parameters of microcapsule phase change cold storage materials prepared with different emulsification speeds.

Core-to-Wall Ratio	Emulsifier Dosage (%)	Crosslinking Agent Dosage (%)	Emulsification Speed (rpm)	Elicitor Dosage (g)
2.5:1	7	30	1250	0.3
2.5:1	7	30	1500	0.3
2.5:1	7	30	1750	0.3
2.5:1	7	30	2000	0.3

**Table 7:** Enthalpies of microcapsules for low temperatures in the literature.

Core	Wall Material	∆H_m_ (kJ/kg)	References
Tetradecane	Calcium carbonate (CaCO_3_) and silicate (SiO_2_)	71.8/99.9	Phan et al. [36]
Normal alkane mixture	Melamine–urea–formaldehyde	110.0	Chen et al. [37]
Mixture of n-tridecane/n-tetradecane	PMMA	89.63	Ertugral et al. [38]
N-decanol–lauric acid	Polypropylene-reinforced MF	15.4	Li et al. [39]
n-Undecane	PMMA	120.3	This work

**Table 8:** Particle size parameters of n-undecane microcapsules prepared at different emulsification speeds.

Emulsification Speed (rpm)	D (0.5) (μm)	Span	Uniformity
1250	28.64	4.36	1.38
1500	22.47	3.88	1.02
1750	12.68	2.26	0.76
2000	10.25	1.68	0.52

**Table 9:** Thermal performance parameters of microcapsules under different thermal cycles.

Cycle (Times)	Tm (°C)	ΔHm (kJ/kg)	Encapsulation Ratio (%)
0	−25.6	120.3	83.5
50	−25.4	118.5	82.3
100	−25.2	109.4	75.9
200	−25.2	101.7	70.6

## Data Availability

Data are contained within the article.
